# The human complement fragment receptor, C5L2, is a recycling decoy receptor

**DOI:** 10.1016/j.molimm.2008.11.001

**Published:** 2009-03

**Authors:** Anne-Marie Scola, Kay-Ole Johswich, B. Paul Morgan, Andreas Klos, Peter N. Monk

**Affiliations:** a Academic Neurology Unit, University of Sheffield Medical School, Beech Hill Road, Sheffield S10 2RX, UK; b Department of Medical Microbiology, Medical School Hannover (MHH), Carl-Neuberg-Str. 1, Hannover D-30625, Germany; c Department of Medical Biochemistry and Immunology, School of Medicine, Cardiff University, Heath Park Way, Cardiff, UK

**Keywords:** C5a, complement fragment 5a, des Arg, loss of C-terminal Arg residue, CHO, chinese hamster ovary, DMEM, Dulbecco's modified Eagle's medium, DARC, Duffy antigen receptor for chemokines, bt2-cAMP, dibutyryl cyclicAMP, HEK, human embryonic kidney, PMN, polymorphonuclear neutrophils, [Ca^2+^]_i_, intracellular free Ca^2+^, Complement fragment 5a, Anaphylatoxin, Receptor, Inflammation, Decoy

## Abstract

C5L2 is a 7 transmembrane domain receptor for complement fragment C5a that, unlike the classical C5a receptor, C5aR, does not couple to G proteins. However, in mice where C5L2 has been deleted, the response to C5a is altered, suggesting that C5L2 may have a signaling function. In order to investigate whether human C5L2 also has some capacity to transduce signals, we have attempted to produce a signaling competent form of human C5L2 by inserting C5aR sequences at three key G protein activation motifs. However, we detected neither an intracellular Ca^2+^ response nor β-arrestin redistribution in mutated C5L2, suggesting that the potential for G protein coupling is completely absent in this receptor and that, in humans, C5L2 may have functions that are unrelated to signaling. In confirmation of this, we detected constitutive ligand-independent internalization of C5L2 that resulted in the rapid accumulation of C5a and its stable metabolite, C5a des Arg, within the cell with only a small net change in cell surface receptor levels. Internalization was found to be through a clathrin-dependent mechanism that led to the retention and, in cells natively expressing C5L2, the degradation of the ligand within an intracellular compartment. In contrast, the classical C5a receptor, C5aR, internalized ligand much more slowly and a majority of this ligand was released back into the extracellular environment in an apparently undegraded form. These data suggest that a major function of human C5L2 is to remove active complement fragments from the extracellular environment.

## 1 Introduction

Complement fragment 5a (C5a) is a 74-residue polypeptide multifunctional proinflammatory mediator that causes leukocyte chemoattraction and degranulation, increases vascular permeability, and stimulates cytokine secretion (Monk et al., 2007). To date, the only endogenous regulation of this potent peptide is the removal of the terminal arginine residue by carboxypeptidase activities to form C5a des Arg. This plasma-stable metabolite is still biologically active but possesses a different spectrum of activities compared to intact C5a (Eglite et al., 2000). The classical receptor for C5a (C5aR) is a member of the G protein-coupled receptor superfamily (Boulay et al., 1991; Gerard and Gerard, 1991) and has high affinity for intact C5a but 10–100-fold lower affinity for C5a des Arg. Recently, the orphan receptor *GPR77* has been identified as a second C5a receptor and was named C5a like receptor 2 (C5L2) (Cain and Monk, 2002; Ohno et al., 2000; Okinaga et al., 2003). C5L2 binds C5a with nearly the same affinity as the C5aR but has a 20-fold higher affinity for C5a des Arg compared to C5aR (Cain and Monk, 2002; Scola et al., 2007). C5L2 has also been proposed as a receptor for other complement fragments, C3a and C3a des Arg, that control the level of triglyceride synthesis in adipocytes (Kalant et al., 2005) and hormone production in the pituitary (Francis et al., 2003), although this remains controversial (Johswich et al., 2006; Okinaga et al., 2003). Studies have found that although C5L2 has the conventional seven transmembrane domain structure of a GPCR, there is no productive coupling to G-proteins (Cain and Monk, 2002; Johswich et al., 2006; Ohno et al., 2000; Okinaga et al., 2003). This is possibly due to the lack of the highly conserved DRY (D131LC in C5L2) and NPXXY (N287PLMF) motifs, found in the third and seventh transmembrane domains, respectively, and a truncated third intracellular domain. Mutation of leucine132 to arginine in human C5L2 has been reported to increase coupling to Gα16 in co-transfected HEK cells (Okinaga et al., 2003). However, there have been reports of signaling by C5L2 that might not involve G protein activation: in HEK cells, transfected C5L2 can stimulate β-arrestin translocation (Kalant et al., 2005); in mouse neutrophils, C5L2 can modulate the signaling activities of C5aR and the related receptor for C3a, C3aR (Chen et al., 2007) and in a mouse model of sepsis, C5L2 appears to have as important a pro-inflammatory role as C5aR (Rittirsch et al., 2008).

The apparent ability of C5L2 to bind anaphylatoxins without stimulating G protein activation has led to the suggestion that C5L2 may have a role as an anaphylatoxin decoy receptor and recent experimental studies offer some support to this view. Rat polymorponuclear neutrophils (PMN) stimulated with C5a and LPS in the presence of C5L2 blocking antibody produced dramatically increased levels of IL-6 compared to control (Gao et al., 2005). PMN from C5L2^−/−^ mice show increased responses to both C5a and C5a des Arg, with an increased influx of PMN into the lung and higher levels of TNF-α and IL-6 when compared to *wt*-mice in a model of pulmonary immune complex injury (Gerard et al., 2005). Further, a comprehensive study of sepsis patients found a higher level of C5L2 content in PMN obtained from patients who survived the observation period compared to patients who failed to survive; low C5L2 expression correlated with sepsis-induced multi-organ failure (Huber-Lang et al., 2005). The biological function of a decoy receptor is to compete with a signaling receptor for ligand and/or to sequester ligand and target it for degradation (Mantovani et al., 2003). Decoy receptors are now recognized as a strategy for negatively regulating the primary inflammatory chemokines (reviewed in Locati et al., 2005). Several chemokine decoy receptor have been identified: US28, CCX CKR, DARC and D6, which possess a set of properties that make them ideally adapted to act as decoy receptors. All have the seven transmembrane domain structure typical of G-protein coupled receptors, most lack the DRY motif and none have been found to couple to G-proteins. In addition, some decoy receptors have been shown to undergo constitutive recycling with a consequently high proportion of intracellular receptors, to be highly efficient at internalizing ligand and to target ligand for degradation. Thus, a receptor with many or all of these properties can be defined as a decoy receptor.

Previous studies using both antibody blockade and receptor knock-out mice have suggested that C5L2 is important in the regulation of C5a and C5a des Arg and that it could act as a ‘decoy’ receptor for these ligands whereas other studies indicate a signaling function for this receptor. The main aim of this investigation is to determine if C5L2 has properties similar to the more fully characterized ‘decoy’ chemokine receptors D6, DARC and US28: undetectable G protein-dependent signaling, a primarily intracellular localization, constitutive internalization, net transport of ligand into the cell and degradation of internalized ligand.

## 2 Methods and materials

### 2.1 Cell culture

RBL-2H3 and Chinese hamster ovary (CHO) cells were routinely cultured in Dulbecco's modified Eagle's medium (DMEM) plus 10% (v/v) fetal calf serum supplemented with 500 mg/l G-418 for transfected cells, at 37 °C, 5% CO_2_. HL-60 cells were cultivated under the same conditions in RPMI 1640 medium supplemented with 10% (v/v) fetal calf serum. For induction of HL-60 into more mature monocyte-like cells, 1 mM of dibutyryl-cyclic AMP (bt2-cAMP) was added to the medium for 3 days. HEK cells stably transduced with C5aR or C5L2 (Johswich et al., 2006) were cultured in DMEM/Ham's F-12 medium plus 10% fetal calf serum supplemented with 800 mg/l G-418. HeLa cells were cultured in modified Eagle's medium supplemented with 10% fetal calf serum, 2 mM l-glutamine, 0.1 mM non-essential amino acids and 1 mM sodium pyruvate.

### 2.2 Isolation of PMN

Human blood was withdrawn from healthy volunteers and the PMN cell fraction was isolated using Polymorphoprep (Axis-Shield) following the manufacturer's advice.

### 2.3 Receptor cloning and expression

Constructs were cloned into pEE6 (Celltech) and transfected into CHO or RBL cells by standard electroporation protocols. After selection in G418, homogenous populations of cells were produced by two rounds of fluorescence-activated cell sorting using a rabbit polyclonal antiserum that recognizes the N-terminal sequence of human C5L2 on a BD Biosciences Aria flow cytometer. HEK cells expressing C5L2 were generated as previously described (Johswich et al., 2006).

### 2.4 Production of antibodies and recombinant ligands

Antiserum against a synthetic peptide analog of the N terminus of human C5L2 (1–32) was prepared as described previously (Kalant et al., 2003). Recombinant C5a and C5a des Arg were synthesized as described previously (Paczkowski et al., 1999). For transfected cells, anti-C5aR antibody W17/1 and a mouse monoclonal that recognizes the hemagglutinin-tag fused to the N-terminus of C5L2 were purchased from Serotec and Sigma, respectively.

### 2.5 Construction of mutants of human C5L2

Substitution mutants DRY (D131LC – DRF), NPXXY (N287PMLF – NPMLY) and a double mutant DRY/NPXXY were made using Stratagene QuikChange kits according to the manufacturer's instructions using HA-C5L2-pEE6 as template and were authenticated by DNA sequencing. C5L2 was further manipulated to produce a chimeric receptor with the intracellular loop 3 of C5aR, substituting residues 223–237 of hC5L2 (LLCWAARRCRPLGTA) with 226–243 of hC5aR (LLRTWSRRATRSTKTLKV). Finally, a triple mutant receptor (+++) was produced containing both substitution mutations and the transplanted loop, using the chimeric C5L2 pEE6 as template for the reaction. C-terminal GFP tagged receptors were made using Invitrogen CT-GFP Fusion TOPO^®^ Expression Kit according to manufacturer's instructions. All modifications were authenticated by DNA sequencing.

### 2.6 Immunocytochemistry

Cells were plated on to poly-l-lysine treated coverslips in a 24 well plate, at 0.75 × 10^5^ cells per coverslip. Next day, cells were washed with PBS and 1 ml of cell buffer (PBS + 1 μM Ca^2+^, 0.1% BSA) added to well and placed at 37 °C for 10 min. Cells were then treated with either buffer or 10 nM C5a for 15 min. Coverslips were washed thoroughly three times after which 4% paraformaldehyde was added for 15 min followed by a further three washes in PBS and then cells were permeabilized with 0.1% Triton-X 100 for 5 min. After three more washes in PBS and blockade with 5% FCS for 1 h, antibodies (anti-N-terminus of C5aR or HA-antibody for C5L2) diluted in 5% FCS were incubated for 1 h followed by three washes in PBS followed by incubation with anti-mouse Ig-FITC (Sigma). For detection of β-arrestin, we used rabbit polyclonal antibody supplied by Dr Robert Lefkowitz (Duke University) against β-arrestin 1 (C-terminus), and anti-rabbit-IgG-FITC (Dako) to determine translocation of arrestin. Coverslips were mounted onto microscope slides with mountant (50% glycerol, 0.01% sodium azide).

### 2.7 Intracellular free calcium assay

RBL cells expressing C5aR, C5L2 or mutant C5L2 were loaded with 1 μM Fluo4-AM (Sigma) plus 0.04% (w/v) Pluronic-F27 (Sigma) at room temperature for 1 h. Cells were then washed in PBS and reconstituted in cell buffer. The assay was carried out on a BD Bioscience FACS Calibur; background fluorescence was recorded for 10 s before the addition of C5a or C5a des Arg and the increase in fluorescence recorded for a further 70 s.

### 2.8 Constitutive internalization

CHO and RBL cells expressing C5aR or C5L2 were resuspended at 2 × 10^6^ ml^−1^ in PBS. Anti-receptor antibody was added and allowed to bind on ice for 1 h. Cells were washed four times with ice-cold PBS and then resuspended in cell buffer before incubation at 37 °C. At appropriate time points aliquots were removed and quenched in BBN on ice. After all time points were collected, the cells were centrifuged at 4 °C and washed with ice-cold PBS before incubation with FITC-labeled secondary antibody for 1 h on ice. Cells then washed twice in ice-cold BBN and cell-associated fluorescence measured by flow cytometry. Hyptonic shock/K^+^ depletion was performed as described (Hansen et al., 1993). Cell surface receptor expression was calculated relative to zero time (=100%). Comparison of curve fitting (GraphPad Prism) was performed using a null hypothesis of straight line fit; the preferred line fit is shown.

### 2.9 Receptor recycling assay

RBL cells transfected with C5aR and C5L2 were resuspended at 4 × 10^6^ ml^−1^ in PBS, anti-receptor antibody was added and allowed to bind for 1 h on ice. Cells were then washed four times in ice-cold PBS and then resuspended in DBH (DMEM + 0.2% BSA with 12.5 mM HEPES) pH 7.4. Cells were incubated at 37 °C for 30 min to allow antibody internalization then stored on ice. Cells were harvested by centrifugation and surface antibody stripped by brief incubation in DBH pH 3 on ice. Cells were then washed three times in ice-cold PBS and then resuspended in DBH pH 7.4. This was then split into 2 aliquots, one incubated at 4 °C and one at 37 °C. At appropriate time points, samples were removed and quenched in BBN on ice. Cells were harvested and incubated with anti-receptor antibodies for 1 h on ice, then washed twice and resuspended in BBN for flow cytometry. Surface receptor expression was calculated as the ratio between the expression levels of cells incubated at 37 °C with that of the cells incubated at 4 °C.

### 2.10 Radioligand uptake experiments

CHO or RBL cells transfected with C5aR or C5L2 were resuspended at 2 × 10^6^ cells/ml in PBS and 0.1 nM ^125^I-C5a or 100 nM ^125^I-C5a des Arg incubated with cells at 4 °C for 1 h. HeLa cells and bt2-cAMP differentiated HL-60 cells were resuspended at 10^7^ cells per ml in PBS for preincubation with 10 μM of the C5aR antagonist AcF[OPdChaWR] (Finch et al., 1999) and (where indicated) 100 μM of phenylarsine oxide or 50 μg/ml nystatin for 10 min at room temperature. The cells were washed twice in ice-cold PBS and then resuspended in DBH (HeLa and HL-60 cells were resuspended at 5 × 10^6^ cells/ml; in corresponding samples the buffer was supplemented with 50 μg/ml nystatin) before incubation with radioligand. At this point, samples were removed and stored in BBN on ice and the remainder transferred to 37 °C. At appropriate time points, aliquots were removed and placed in BBN on ice. Cells were then harvested by centrifugation and washed in ice-cold DBH pH 3. Radioactivity contained in the cell pellets was determined using a Gamma counter (LKB Wallac 1271). Results were calculated as the ratio of radioactivity in the acid-washed cell pellet to initial radioactivity in cells incubated at 4 °C.

### 2.11 Competitive ^125^I-C5a binding assay

HL-60 cells were fixed for 10 min with Cellfix (Becton Dickinson) and permeabilized for 3 min with 0.1% saponin in PBS or treated with PBS only prior to incubation for 10 min with 10 μM of the C5aR antagonist AcF[OPdChaWR] at room temperature. Ligand binding assays of PMN and permeabilized or intact HL-60 cells were performed as previously described (Johswich et al., 2006). Briefly, cells were incubated over night at 4 °C with 0.1 nM ^125^I-C5a and increasing concentrations of unlabeled C5a. The cells were washed twice and bound radioactivity was measured using a TopCount NXT (Canberra Packard) counter. *B*_max_ was calculated with the LIGAND program (Bio-Soft).

### 2.12 Ligand fate assay

This was performed largely as described previously (Weber et al., 2004). Briefly, cells were treated as for the radioligand uptake experiments, except that aliquots were removed after 0, 20, 60 and 120 min at 37 °C, and quenched with BBN on ice. Cells were then pelleted by centrifugation and the supernatant removed. The cell pellet was retained to determine radioactivity. Trichloroacetic acid (TCA) was added to the supernatants to a final concentration of 12.5% and precipitation allowed to occur on ice for 15 min. After this time, the precipitates were collected by centrifugation at 12,000 × *g* for 15 min and the supernatants removed and retained for determination of radioactivity (non-precipitated). The precipitated protein pellet was washed in ice-cold acetone and centrifuged at 12,000 × *g* for 5 min. The supernatant from this wash was pooled with non-precipitated protein from the previous centrifugation. The radioactivity present in the cell pellet, precipitated supernatant protein and non-precipitated supernatants were counted using using an LKB Wallac, 1271 gamma counter.

## 3 Results

### 3.1 G protein-coupled signaling by C5L2 is repressed by multiple mechanisms

**Fig. 1:**
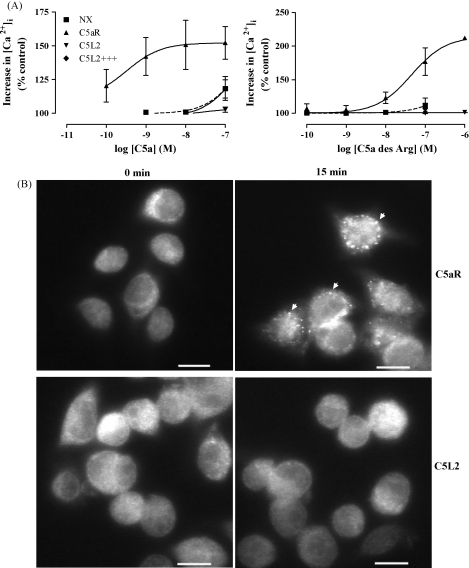
C5a does not stimulate increases in intracellular Ca^2+^ or translocation of β-arrestin in RBL cells transfected with C5L2. (A) Fluo4-AM loaded RBL cells transfected with C5aR, wild type C5L2, C5L2 with C5aR-like sequences at three key signaling motifs (C5L2+++) or untransfected controls (NX) were stimulated with C5a (left panel) or C5a des Arg (right panel). Changes in the intracellular Ca^2+^ concentration were measured by flow cytometry as changes in fluorescence integrated over 70 s and shown as a percentage of fluorescence in unstimulated cells. Results shown are means of three separate experiments performed in duplicate ± S.E.M. (B) Transfected RBL cells adhered to coverslips were treated with 100 nM C5a for 15 min, fixed, permeabilized, incubated with antibody specific for β-arrestin 1. Arrows show intracellular accumulation of β-arrestin 1 in C5aR-transfected cells. Bar equal to 10 μm.

Although several studies have failed to detect signaling by C5L2, others have demonstrated some capacity for signal transduction (Cain and Monk, 2002; Chen et al., 2007; Johswich et al., 2006; Kalant et al., 2005; Lee et al., 2008; Okinaga et al., 2003; Rittirsch et al., 2008). It is possible that C5L2 has a small capacity to transduce ligand-binding signals that is difficult to detect due to attenuation by the lack of highly conserved motifs (DRY in the 3rd transmembrane domain, NPXXY in the 7th transmembrane domain) and/or the short 3rd intracellular loop. To investigate this possibility, we made a mutant of C5L2 (C5L2+++) in which the motifs from the efficiently coupled C5a receptor, C5aR were substituted for the C5L2 sequence: D131LC-D131RF; N287PMLF–N287PMLY and the entire third intracellular loop sequence of C5aR. Signaling capacity was measured using the highly sensitive fluorescent intracellular free Ca^2+^ ([Ca^2+^]_i_) assay. When C5aR expressing RBL cells were stimulated with C5a (from 0.1 nM) there was a dramatic increase in fluorescence (Fig. 1A, left panel). In contrast, RBL cells transfected with wild type C5L2 or the mutant C5L2+++ showed no increase in fluorescence upon stimulation with concentrations of C5a of up to 100 nM, suggesting that no G protein-coupled signaling was occurring (Fig. 1A). Similarly, C5a des Arg could stimulate an increase in [Ca^2+^]_i_ in C5aR-transfected RBL cells at concentrations of 10 nM and above but no changes were detectable in C5L2 or C5L2+++ transfected cells at any concentration used (Fig. 1A, right panel). Constructs of C5L2 with individual motifs mutated to their equivalents in C5aR had the same responses to both C5a and C5a des Arg (data not shown). A previous report showed some coupling of a C5L2 mutant (D131RC) to Gα16 in transiently co-transfected HEK cells (Okinaga et al., 2003). Signaling here may have been detected due to very high levels of receptor expression and also because the promiscuous Gα16 is less discriminating than the endogenous Gαi in RBL cells. Nevertheless, these results confirm that signaling by human C5L2 is highly repressed and there are multiple structural features that prevent Gαi activation by C5L2. β-Arrestin is a cytosolic protein that mediates the desensitization and internalization of GPCR and has also been shown to mediate G protein-independent signaling by 7 transmembrane domain receptors (reviewed by Defea (2008)). It has been found to translocate from the cytosol to the plasma membrane and associate with GPCR only once the receptors have been bound by their ligands and phosphorylated by GPCR kinases. In keeping with our previous findings (Kalant et al., 2005), there was obvious ligand-induced translocation of β-arrestin in RBL cells expressing human C5aR (Fig. 1B). β-Arrestin was seen throughout the cytosol prior to ligand stimulation but appeared to accumulate in granular structures after 15 min stimulation with 100 nM C5a. In contrast to previous studies that showed distinctive translocation of exogenous GFP-tagged β-arrestin in co-transfected HEK cells after stimulation with C5a (Kalant et al., 2005), we found there was no obvious translocation of β-arrestin 1 after C5a stimulation of human C5L2 (Fig. 1B). Human C5L2 is unable to induce the translocation of endogenous β-arrestin 1 in the RBL cell line.

### 3.2 C5L2 is primarily an intracellular protein

**Fig. 2:**
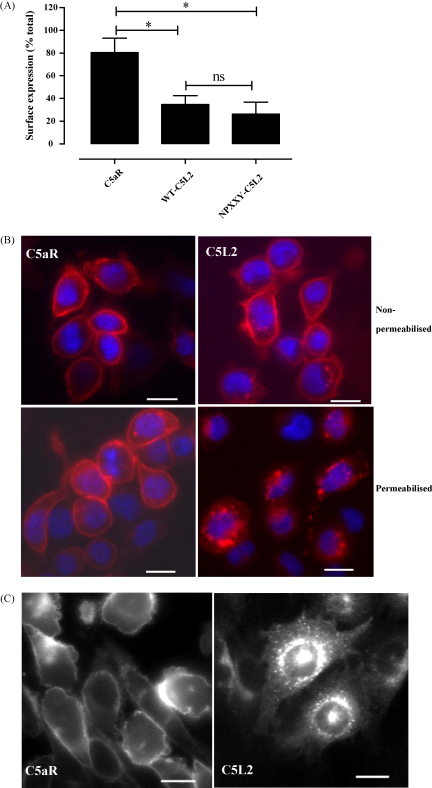
C5L2 has a primarily intracellular location in transfected RBL cells. (A) The expression pattern of receptor in RBL cells transfected with C5aR, wild type or mutant C5L2 (NPXXY) was measured by incubating permeabilized and intact cells with anti-receptor antibodies. The percentage surface expression was calculated as the ratio of antibody binding to intact cells to that in permeabilized cells. The data shown are the mean ± S.E.M. of three to four separate determinations performed in duplicate. Significantly different by ANOVA with Bonferroni post-test, *p < 0.05; ns: not significant. (B) Intracellular and surface receptor expression was visualized in RBL cells transfected adhered to coverslips, incubated with antibody specific for the N-terminus of C5aR or the N-terminal HA tag of C5L2 with or without a permeabilization step; nuclei are counter-stained in blue. Bar equal to 10 μm. (C) CHO cells transfected with GFP tagged constructs of C5aR or C5L2 mutants were adhered to coverslips. Bar equal to 10 μm.

The intracellular location of C5aR and wild type C5L2 were determined initially by flow cytometry of permeabilized and non-permeabilized transfected RBL cells using monoclonal antibodies recognizing N-terminal epitopes (Fig. 2A). Similar results were obtained when a polyclonal anti-receptor anti-serum was used to detect C5L2 (data not shown). Only 20% of C5aR was found within cells whereas >60% of C5L2 was intracellular, despite the cell-sorting of C5L2-transfected cells on the basis of surface expression of the receptor. The NPXXY motif has been associated with receptor trafficking (Barak et al., 1994) and so we investigated whether the lack of this motif in wild type C5L2 was causing intracellular retention. However, the C5L2 mutant NPXXY (N287PMLF–N287PMLY) had a very similar distribution to wild type C5L2 (Fig. 2A). Receptor distribution was confirmed by fluorescence microscopy on unpermeabilized and on permeabilized RBL cells. Although there appear to be comparable levels of surface expression of both receptors (Fig. 2B, top panels), the majority of C5L2 in permeabilized cells was clearly in granular structures in the cytoplasm (Fig. 2B, lower panels). Receptor constructs were made with GFP fused to the C-terminus and also used to study the cellular localization of this receptor (Fig. 2C). When expressed in CHO cells, these tagged receptors had very similar distributions to those observed using antibody labeling (Fig. 2A and B), with C5L2 predominantly inside the cell. However, this pattern of expression could be an artifact due to the excessive protein production in transfected cells. To test this, HL-60 cells were induced to differentiate into a more mature monocyte phenotype by incubation with bt2-cAMP, a treatment known to stimulate the endogenous expression of both C5aR and C5L2 (Johswich et al., 2006). These cells were found to express C5L2 in primarily intracellular locations as determined using a competitive, C5L2-restricted, ^125^I-C5a binding assay on intact or saponin permeabilized cells. In the presence of the C5aR antagonist AcF[OPdChaWR], only about 20% of the ^125^I-C5a binding to C5L2 occurred at the surface (6584 ± 1840 C5L2 molecules per cell at the surface from a total of 27735 ± 10782 per cell; mean ± standard error, *n* = 3). Although the polyclonal antibodies used in this study recognize ectopic C5L2 in transfected cells, they bind only poorly to natively expressed C5L2 in HL-60 cells or PMN (data not shown). However, a monoclonal antibody that recognizes native C5L2 (Lee et al., 2008) has been used to demonstrate the presence of predominantly intracellular expression of C5L2 in human monocytes.^2^ Thus, overexpression of C5L2 in transfected cells does not account for the observed intracellular localization.

### 3.3 Internalization of C5L2 is constitutive

**Fig. 3:**
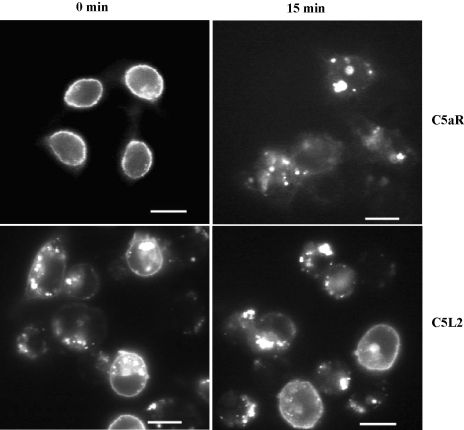
C5L2 does not undergo ligand-dependent internalization in transfected RBL cells. Transfected RBL cells were incubated with 100 nM C5a at 37 °C for 15 min and then fixed and permeabilized. Receptor was visualized with antibody specific for the N termini of C5aR or the N-terminal HA tag of C5L2. Bar equal to 10 μm.

For many GPCR, ligand binding increases the receptor internalization rate, manifested as a reduction in cell surface receptor levels (Marchese et al., 2008). However, previous reports by our group and others (Cain and Monk, 2002; Okinaga et al., 2003) have shown that, unlike C5aR, C5L2 does not undergo ligand-induced internalization upon stimulation with ligand. The lack of internalization or re-distribution of C5L2 was confirmed by fluorescence microscopy of transfected RBL cells, where the ligand-induced accumulation of C5aR in discrete granular structures is obvious (Fig. 3). In contrast, C5L2 was primarily located in intracellular granular structures prior to the addition of ligand. Again, NPXXY-C5L2 behaved identically to the wild type receptor (data not shown).

**Fig. 4:**
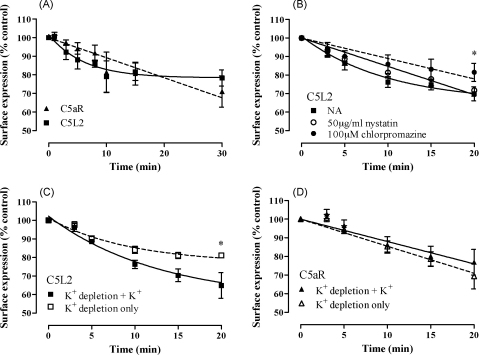
Different mechanisms are involved in the turnover of surface C5aR and C5L2 in transfected RBL cells. (A) RBL cells transfected with C5aR or C5L2 were incubated with monoclonal antibodies specific for the N-terminus of C5aR or the N-terminal HA tag of C5L2 for 1 h at 4 °C then washed extensively. Cells were warmed to 37 °C for the indicated times before quenching in ice-cold buffer. Surface antibody was quantified by flow cytometry. Data shown are mean ± S.E.M. from six experiments. (B) RBL cells transfected with C5L2 were incubated with buffer only (NA), 50 μg/ml nystatin or 100 μM chlorpromazine prior determination of surface anti-C5L2 antibody. Data shown are mean ± S.E.M. from three to six separate experiments; (C and D) RBL cells transfected with C5aR (C) or C5L2 (D) were incubated in DMEM/H_2_O (1:1) for 5 min and then incubated in K^+^ free buffer for 30 min at 37 °C prior to incubation with antibodies specific for the N-terminal HA tag of C5L2 (C) and the N terminus of C5aR (D) for 1 h at 4 °C in buffer with K^+^ (shaded symbols) or without K^+^ (open symbols). Excess antibody was removed by extensive washing and then cells incubated at 37 °C in buffer with K^+^ (shaded symbols) or without K^+^ (open symbols) for the times indicated. After quenching, surface antibody was quantified by flow cytometry. Data shown are mean ± S.E.M. from three to seven experiments. Significantly different from control cells at the same time-point by two-way ANOVA with Bonferroni post-test; *p < 0.05. In all cases, data fitting to straight line (null hypothesis) or one-phase decay models was compared. Data is shown fitted to a straight line unless null hypothesis rejected, *p* < 0.05.

Most GPCR that endocytose ligand undergo ligand-induced internalization, whereby both receptor and ligand are taken into the cell and targeted to the endosomal pathway (Marchese et al., 2008). Alternatively, a small number of receptors undergo constitutive internalization. This is the case for the D6 chemokine receptor, the net surface expression of which does not change, despite rapid ligand uptake (Weber et al., 2004). The current model of this receptor is that it undergoes constitutive trafficking from the cell surface in a clathrin-dependent manner (Bonecchi et al., 2008; Galliera et al., 2004; McCulloch et al., 2008; Weber et al., 2004), with bound ligand ‘hitching’ a ride into the cell. We set out to determine whether C5L2 also undergoes constitutive recycling, firstly by investigating the receptor turnover from the surface of transfected RBL cells by labeling receptor with antibody and following loss of antibody over time. It can be seen that both C5L2 and C5aR show a steady loss of receptor over time (Fig. 4A) although the rate of loss of C5L2 from the surface decreases at 10–15 min whereas C5aR expression falls at a constant rate. Comparison of curve fits indicates that the rate of C5L2 internalization is one-phase exponential decay (null hypothesis of straight line fit rejected, *p* = 0.001) whereas the rate of C5aR internalization was constant (null hypothesis of straight line fit not rejected, *p* = 0.42). To determine the mechanism involved in the removal of receptors from cell surface, we used nystatin, to inhibit caveolin-dependent processes (Stahl and Mueller, 1995) and chlorpromazine pre-treatment or hypotonic shock/depletion of extracellular K^+^, to inhibit clathrin-mediated endocytosis (Subtil et al., 1994). Chlorpromazine (Fig. 4B) or K^+^ depletion treatment (Fig. 4C) significantly inhibited the loss of C5L2 from of the cell surface relative to control (where K^+^ is added back after hypotonic shock) whereas nystatin had no effect (Fig. 4B). In contrast, K^+^ depletion had no effect on C5aR (Fig. 4D), suggesting that C5L2 endocytosis is clathrin-dependent whereas the apparent constitutive loss of C5aR from the surface occurs by a distinctly different mechanism.

### 3.4 Internalized C5L2 is rapidly re-expressed

**Fig. 5:**
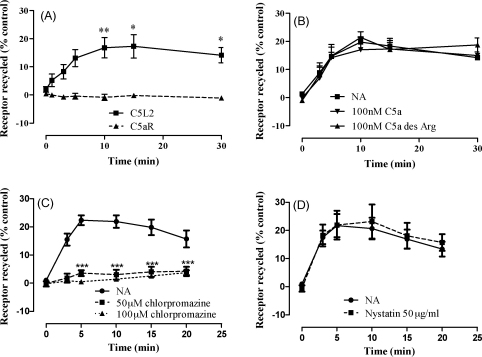
C5L2 but not C5aR undergoes constitutive recycling in transfected RBL cells. Transfected RBL cells were incubated with antibodies specific for the N termini of C5aR and the N-terminal HA tag of C5L2 for 1 h at 4 °C. Excess antibody was removed by extensive washing with ice-cold PBS and then cells warmed to 37 °C for 30 min. Cells were then washed in ice-cold acid medium before incubation at 37 or 4 °C for the indicated times in the presence of buffer alone (A), 100 nM C5a or C5a des Arg (B), the indicated concentrations of chlorpromazine (C) or nystatin (D). After quenching, surface antibody was detected by flow cytometry. The results are shown as a percentage increase in receptor expression at 37 °C relative to cells maintained at 4 °C and are the mean ± S.E.M. of three to six separate experiments performed in duplicate. Significantly different from controls or C5aR at the same time-point by two-way ANOVA with Bonferroni post-test; *p < 0.05, **p < 0.01, ***p < 0.005.

To determine the fate of endocytosed receptors, the protocol used to detect receptor endocytosis was modified with the addition of an acid wash step to remove antibodies from the surface of RBL cells prior to further incubation at 37 °C. Therefore reappearance of antibody at the cell surface would indicate the recycling of receptors (Weber et al., 2004). It can be clearly seen that there is no reappearance of anti-C5aR antibody after acid washing (Fig. 5A). In contrast, there is a significant reappearance of C5L2 antibody over time, with peak reappearance (17 ± 4%) occurring at 15 min (Fig. 5A), suggesting that the receptor undergoes constitutive recycling. A similar level of recycling was also observed in CHO cells transfected with C5L2 (data not shown). The presence of ligand did not alter the ability of chemokine decoy receptor, D6, to undergo constitutive recycling (Weber et al., 2004). In keeping with this, our studies showed that there was no effect on the ability of C5L2 to undergo constitutive recycling either in the presence of high levels of C5a or C5a des Arg (Fig. 5B). Incubation of the cells with the clathrin inhibitor, chlorpromazine, caused significant inhibition of recycling (Fig. 5C) but no effects were seen with the caveolin inhibitor, nystatin (Fig. 5D).

### 3.5 Ligand internalized by cells transfected with C5L2 is retained within the cell

**Fig. 6:**
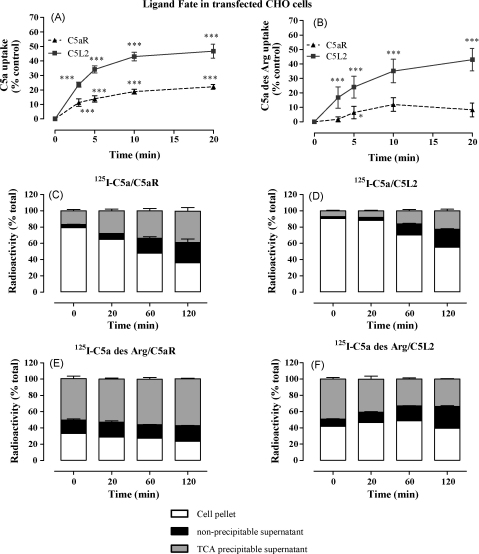
C5L2 in transfected CHO cells is more efficient than C5aR at internalizing and retaining C5a and C5a des Arg but ligand degradation does not occur in these cells. (Panels A and B) *Ligand internalization*: transfected cells expressing equal levels of receptor were loaded with ^125^I-C5a (A) or 100 nM ^125^I-C5a des Arg (B) at 4 °C for 1 h, then extensively washed and warmed to 37 °C for the indicated times. Cells were then washed in either ice-cold PBS or acidic medium to strip cell-surface ligand. Results are shown as percentage of radioactivity in acid-washed cells compared to cells washed in PBS and are the mean ± S.E.M. of 8–10 separate experiments performed in duplicate. (C–F) *Ligand fate*: cells expressing equal levels of receptor were loaded with 0.1 nM ^125^I-C5a (C and D) or 100 nM ^125^I-C5a des Arg (E and F) at 4 °C for 1 h, then extensively washed and shifted to 37 °C for the specified times. Cells were harvested and the supernatant subjected to TCA precipitation. The results are shown as a percentage of total radioactivity per sample found in cell pellet, precipitated and non-precipitated protein from supernatant and are the mean ± S.E.M. of four separate experiments performed in duplicate. Significantly different from zero-time by two-way ANOVA with Bonferroni post-test *p < 0.05; ***p < 0.005, Student's *t*-test.

One feature of the chemokine decoy receptors is their ability to rapidly endocytose ligand. Further, ligands internalized by decoy receptor D6 are targeted for rapid degradation unlike ligands of the non-decoy receptor, CCR5 (Weber et al., 2004). We were interested to see if C5L2 also had this ability. Using ^125^I-C5a and C5a des Arg, we carried out ligand-uptake experiments, using an acid wash step to remove cell surface ligand. In transfected CHO cells which expressed high (and similar) levels of surface C5aR and C5L2, rapid internalization of both ligands was observed; this was significantly higher on cells expressing C5L2 as compared to cells expressing C5aR (Fig. 6A and B). There was almost no uptake of C5a des Arg by C5aR, probably due to the lower affinity of C5aR for this protein. Transfected RBL cells also showed C5L2-dependent uptake of C5a/C5a des Arg, although to a lesser extent, most likely due to lower receptor expression levels (data not shown). We also investigated whether the ligand endocytosed by C5L2 became degraded, estimated by the appearance of TCA-soluble radioactivity. As expected, there were significantly higher levels of ^125^I-C5a retained in the cell pellet of C5L2-transfected CHO cells (∼20% at 20, 60 and 120 min (*p* < 0.001)) compared to C5aR. However there was no significant increase in the degradation of ^125^I-C5a or ^125^I-C5a des Arg over time between C5aR and C5L2 (Fig. 6C–F). Ectopic expression of C5L2 appears, in this case, to confer the ability to store ligand internally but not to cause its degradation.

**Fig. 7:**
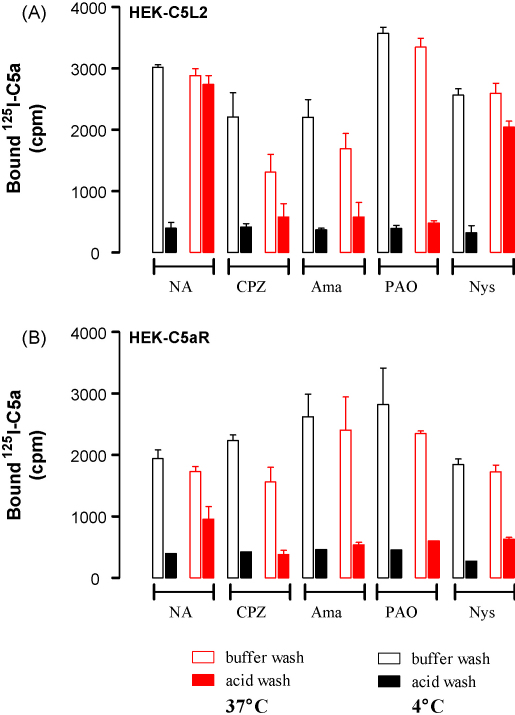
Ligand uptake in HEK cells transfected with C5L2 is sensitive to inhibitors of clathrin-mediated internalization HEK cells were transiently transfected with either human C5L2 or C5aR, treated with NA = no addition; Ama = amantadine; CPZ = chlorpromazine; PAO = phenylarsine oxide; Nys = nystatin and incubated with 0.1 nM ^125^I-C5a for 2 h at either 4 or 37 °C, as indicated. The cells were subjected to extensive washing with either buffer at pH7.4 (buffer wash) or pH3 (acid wash). The data shown are from two separate experiments, mean ± S.D.

A separate model system, transiently transfected HEK cells, also showed a temperature-dependent uptake of C5a by C5L2 but not by C5aR (Fig. 7A and B). After 2 h incubation at 37 °C, ∼90% of ^125^I-C5a could not be removed by an acid wash indicating that the majority of the C5a was internalized. The sensitivity of C5a uptake to a series of inhibitors of endocytosis was additionally tested in these cells (Fig. 7A and B): clathrin inhibitors amantadine (Schlegel et al., 1982), chlorpromazine and phenylarsene oxide (Frost and Lane, 1985) lowered the amounts of ^125^I-C5a that was resistant to acid wash whereas caveolin inhibitor nystatin had little effect. Intriguingly, there were decreases in the total binding of ^125^I-C5a at 37 °C to C5L2 in the presence of amantadine and chlorpromazine, suggesting that these inhibitors can decrease basal C5L2 expression.

### 3.6 Ligand internalized by C5L2 in naturally expressing cell lines is retained within the cell and degraded

**Fig. 8:**
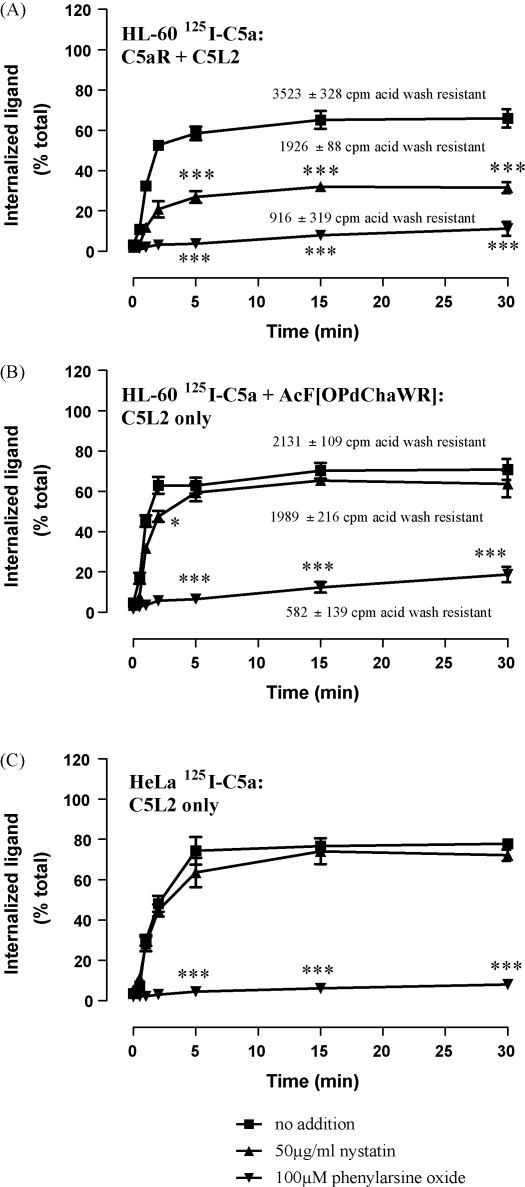
C5L2 in differentiated HL-60 and HeLa cells is responsible for internalizing C5a. Bt2cAMP-differentiated HL-60 and HeLa cells were loaded with ^125^I-C5a at 4 °C for 1 h and (where indicated) 10 μM of the C5aR antagonist AcF[OPdChaWR], 100 μM of phenylarsine oxide or 50 μg/ml nystatin, then extensively washed and shifted to 37 °C for the specified times. Cells were harvested and washed in acidic buffer to strip surface bound ligand. The results show uptake of radioactivity compared to controls incubated at 4 °C and are the mean ± S.E.M. of three to six separate experiments performed in duplicate. The amount of radioactivity actually internalized by HL-60 cells is shown for each condition. Significantly different from control at the same time-point by two-way ANOVA with Bonferroni post-test; *p < 0.05, ***p < 0.005.

It is possible that transfected cells lack aspects of the cellular machinery for the degradation of ligand internalized by C5L2. We tested this possibility in two cell lines: bt2-cAMP differentiated HL-60 cells, which endogenously express C5aR and C5L2, and also in HeLa cells, which only endogenously express C5L2 (Johswich et al., 2006). Using ^125^I-C5a (and removing remaining free ligand by a washing step before cells were warmed up to 37 °C), internalization was rapid and sensitive to the clathrin inhibitor, phenylarsine oxide, in both cell types (Fig. 8A–C). Nystatin moderately inhibited internalization in HL-60 cells (by ∼50%) but no effect of nystatin (<10%) was observed when C5aR was blocked by AcF[OPdChaWR], i.e. when only internalization of ^125^I-C5a by C5L2 was measured. As the total amount of acid wash resistant radioactivity in nystatin-treated cells was almost identical in cells with or without AcF[OPdChaWR] (1989 ± 216 CPM versus 1926 ± 81 CPM per 10^6^ cells), only C5aR internalization seems to be dependent on caveolin in differentiated HL-60 cells. Thus, C5L2-mediated, clathrin-dependent internalization of ligand also occurs in cells that naturally express this receptor.

**Fig. 9:**
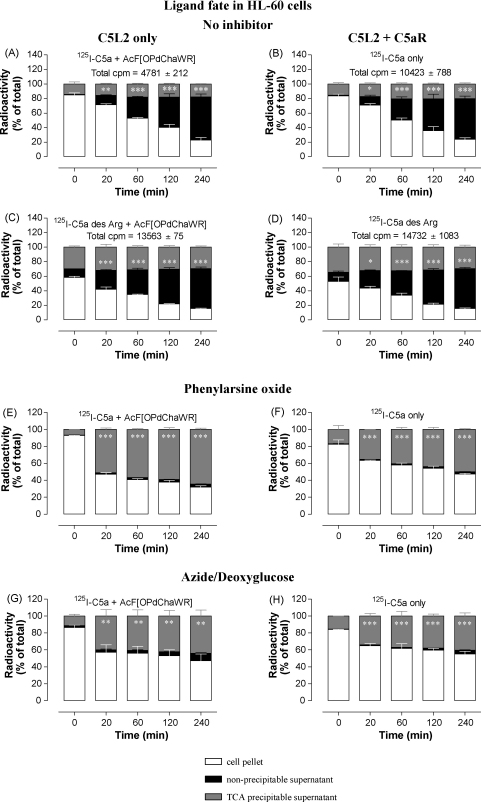
C5L2 in differentiated HL-60 cells is responsible for internalizing, retaining and degrading C5a and C5a des Arg. Bt2cAMP-differentiated HL-60 cells were loaded with ^125^I-C5a or ^125^I-C5a des Arg at 4 °C for 1 h and (where indicated) 10 μM of the C5aR antagonist AcF[OPdChaWR] or 100 μM of phenylarsine oxide or 10 mM sodium azide plus 10 mM 2-deoxy-d-glucose, then extensively washed and shifted to 37 °C for the specified times. Cells were harvested and the supernatant subjected to TCA precipitation. The total radioactivity in each sample is given for A–D and the bars show the percentage of the total radioactivity per sample found in cell pellet, precipitated or non-precipitated protein from supernatant and are the mean ± S.E.M. of two to four separate experiments performed in duplicate. Significantly different from controls at the zero time-point by two-way ANOVA with Bonferroni post-test; ns: not significant, *p < 0.05, **p < 0.01, ***p < 0.005.

**Fig. 10:**
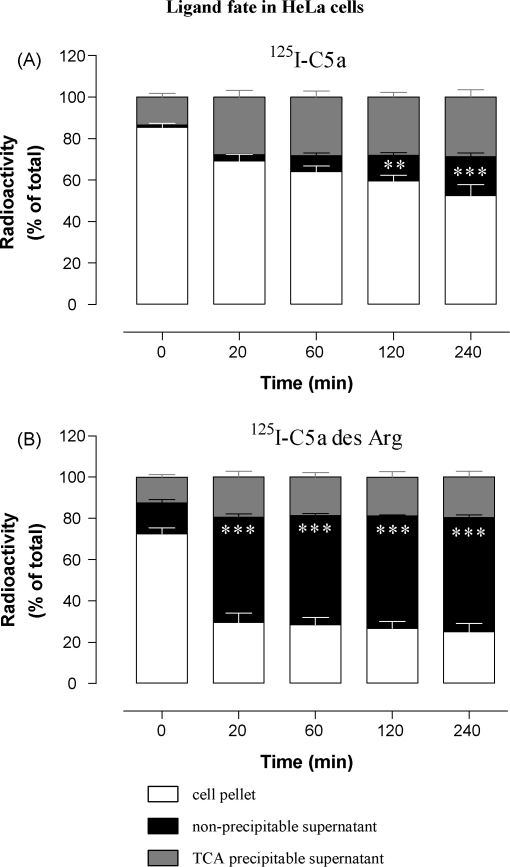
C5L2 in HeLa cells is responsible for internalizing, retaining and degrading C5a and C5a des Arg. HeLa cells were loaded with ^125^I-C5a or ^125^I-C5a des Arg at 4 °C for 1 h, then extensively washed and shifted to 37 °C for the specified times. Cells were harvested and the supernatant subjected to TCA precipitation. The results are shown as a percentage of the total radioactivity per sample found in cell pellet, precipitated and non-precipitated protein from supernatant and are the mean ± S.E.M. of two to three separate experiments performed in duplicate. Significantly different from controls at the zero time-point by two-way ANOVA with Bonferroni post-test; ns: not significant, *p < 0.05, **p < 0.01, ***p < 0.005.

A clear degradation of both C5a and C5a des Arg was also observed in both differentiated HL-60 and HeLa cells (Figs. 9 and 10), measured as the increase of TCA-soluble radioactivity with time. In HL-60 cells, 50–60% of ligand was apparently degraded after 240 min and, again, this effect was not inhibited by C5aR antagonist, strongly suggesting that ligand degradation occurs after uptake by C5L2 (Fig. 9A–D). To prove that cellular uptake was indeed a prerequisite for degradation of the radioligand, we treated differentiated HL-60 cells with the clathrin inhibitor, phenylarsine oxide, which abolished ligand degradation (Fig. 9E and F) and led to the release of intact ligand back into the supernatant. When the cells underwent ATP depletion by treatment with sodium azide and 2-deoxy-d-glucose, they also lost their capacity to degrade ligand (Fig. 9G and H) and again released undegraded ligand. ^125^I-C5a binding was normal (data not shown) in the presence of all of these inhibitors. Viability seemed to be affected to only a small extent by these treatments, as measured by trypan blue exclusion: 83.4 ± 0.67% (88.2 ± 1.82%) of azide/2-deoxy-d-glucose and 60.6 ± 5.35% (89.5 ± 1.68%) of phenylarsine oxide treated cells excluded dye (mean ± S.D., viability of untreated control cells in brackets). Importantly, C5a incubated with undifferentiated HL-60 cells which express neither C5aR nor C5L2 showed no degradation at all, even after 120 min (data not shown). HeLa cells internalized ^125^I-C5a rapidly but little degradation was apparent, even after 4 h (Fig. 10A). By contrast, ^125^I-C5a des Arg was both internalized and degraded rapidly, with most radiolabel in a TCA-soluble form within 20 min (Fig. 10B).

**Fig. 11:**
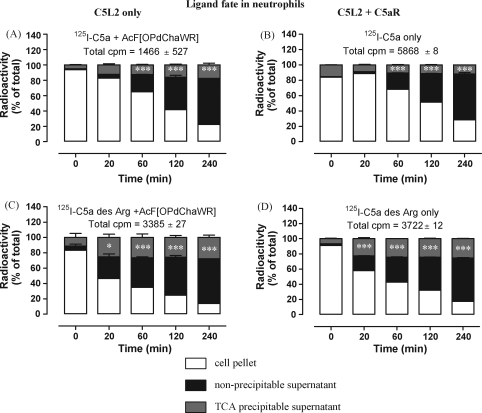
C5L2 in polymorponuclear neutrophils is responsible for internalizing, retaining and degrading C5a and C5a des Arg. Neutrophils were loaded with ^125^I-C5a or ^125^I-C5a des Arg at 4 °C for 1 h and (where indicated) 10 μM of the C5aR antagonist AcF[OPdChaWR], then extensively washed and shifted to 37 °C for the specified times. Cells were harvested and the supernatant subjected to TCA precipitation. The total radioactivity in each sample is given for A–D and the bars show the percentage of the total radioactivity per sample found in cell pellet, precipitated or non-precipitated protein from supernatant and are the mean ± S.E.M. of two separate experiments performed in duplicate. Significantly different from controls at the zero time-point by two-way ANOVA with Bonferroni post-test; ns: not significant, *p < 0.05, ***p < 0.005.

Finally, we wanted to observe C5L2-mediated degradation of C5a/C5a des Arg in primary cells. Human PMN are known to express both C5aR and C5L2 (Okinaga et al., 2003). Using the competitive ^125^I-C5a binding assay, we found that the surface expression of C5L2 in PMN is weak compared to that of C5aR (*B*_max_ = 4617 ± 1373 versus 97306 ± 42658 molecules per cell, mean ± standard error, 4 donors). The percentage of ^125^I-C5a bound to C5L2 (i.e. in the presence of the C5aR antagonist AcF[OPdChaWR]) relative to C5aR varied between donors (range: 2.4–29.7%; median 4.5%), suggesting a wide natural variation in C5L2 expression. As observed in HeLa cells, degradation of ^125^I-C5a des Arg in PMN appears to occur faster than ^125^I-C5a (Fig. 11A–D). Total neutrophil uptake of C5a (comparing absolute CPM values) was decreased by AcF[OPdChaWR] more markedly than in HL-60 cells, C5L2 being accountable for only 25% (Fig. 11A and B). In contrast, there was virtually no effect of C5aR antagonist AcF[OPdChaWR] on the total uptake of ^125^I-C5a des Arg by PMN (Fig. 11C and D), confirming that C5L2 is the main receptor for this processed form of C5a. Taken together, these data suggest that C5L2, in an appropriate cellular milieu, target ligands for degradation.

## 4 Discussion

The potent chemoattractant C5a has a half-life of 2–3 min in the circulation, suggesting the existence of a mechanism for its rapid removal from the circulation (Webster et al., 1982; Weisdorf et al., 1981). The classical G protein-coupled receptor for C5a, C5aR, has been proposed to form a major part of this mechanism (Oppermann and Gotze, 1994). We hypothesized, however, that the second receptor for C5a, C5L2, which has a limited ability to transduce signals, is also likely to form part of the clearance mechanism. Here, we have used the chemokine scavenging decoy receptor D6 as a template, to determine if C5L2 might play a similar role for complement fragments. D6 binds multiple chemokines but does not couple to G proteins and so cannot transduce signals in the conventional way for a heptahelical receptor. Instead, D6 has been found to be constitutively internalized to an intracellular compartment from the cell surface, carrying with it extracellular ligands. These ligands are then rapidly dissociated from D6 by endosomal acidification, the ligand degraded and the free receptor recycled to the cell surface, thus maintaining a constant level of expression at the plasma membrane. The mechanism of recycling is clathrin and arrestin-dependent, similar to conventional G protein-coupled receptors (Galliera et al., 2004; Weber et al., 2004). The data presented here shows that C5L2 has many similarities with D6. Firstly, there is no evidence of coupling to G proteins, even when sequence motifs found to be critical for G protein-coupling were mutated to their equivalents in C5aR, a receptor that signals strongly in the cell types used. Secondly, both D6 and C5L2 have largely intracellular localizations, unlike the classical signaling receptors C5aR and CCR5 (Weber et al., 2004). Thirdly, like D6, C5L2 does not undergo ligand-induced internalization but does show clear signs of constitutive internalization and recycling: antibodies against an N-terminal tag on C5L2 were internalized and re-expressed in transfected cell lines whereas antibodies against the C5aR N-terminus were not. Although there is a theoretical chance that C5aR recycling was not detected due to a difference in antibodies used, this is unlikely because the antibodies used were both high affinity monoclonal mouse IgG and could be expected to behave in a similar way following internalization. Fourthly, D6 internalization is clathrin-dependent and this is also true for C5L2: inhibitors of clathrin function prevent C5L2 internalization and recycling whereas inhibitors of caveolin function did not. Finally, the internalization by D6 leads to degradation of the ligand and this also occurs (particularly for C5a des Arg) in cells that express endogenous C5L2, such as HeLa, differentiated HL-60 and human blood neutrophils. This did not occur in transfected CHO or RBL cells and this may be due to the lack of the appropriate degradative machinery. Interestingly HeLa cells could only degrade C5a des Arg, perhaps because of the lack of C5aR: C5L2 binds C5a and C5a des Arg by different mechanisms (Scola et al., 2007) and may not be able to release C5a in the appropriate intracellular environment for degradation. C5L2 and C5aR appear to act in concert in producing full cellular responses to C5a in vivo (Chen et al., 2007; Rittirsch et al., 2008) and it is possible that cells expressing both receptors (HL-60, neutrophils) may target both C5a and C5a des Arg for degradation whereas cells that only express C5L2 (HeLa) can target only one ligand, C5a des Arg. C5aR appeared to be responsible for a moderate uptake of C5a in HEK and CHO cells transfected with this receptor; C5aR also displayed a detectable rate of loss from the cell surface in the absence of ligand. However, C5L2 was a much more efficient transporter of C5a in all cases. This is in contrast to chemokine receptors: CCR5 was not found to constitutively internalize at all and D6 by itself could transport multiple chemokines for degradation (Weber et al., 2004). Interestingly, C5aR seems to use a different mechanism for this low level of constitutive internalization compared to C5L2: it is not inhibited by K^+^ depletion in transfected CHO cells but is inhibited by nystatin in differentiated HL-60 cells, suggesting a clathrin-independent, possibly caveolin-dependent mechanism. Internalization of ligand by C5aR does not lead to retention or degradation; instead, ligand appears to be released into the extracellular medium in an apparently intact state. Whether this mechanism has any biological significance is currently unclear.

The data presented here suggests that an important role of human C5L2 is the elimination of C5a des Arg, the primary circulating form of the anaphylatoxin (Bokisch and Muller-Eberhard, 1970; Budzko et al., 1971). It would be expected, therefore, that, like D6, C5L2 would have a primarily anti-inflammatory function *in vivo* (Borroni et al., 2006). This is the case in a mouse model of pulmonary inflammation (Gerard et al., 2005) and also in rat astrocytes (Gavrilyuk et al., 2005) but several groups have recently reported a pro-inflammatory role for mouse C5L2, albeit without evidence of the involvement of G proteins (Chen et al., 2007; Rittirsch et al., 2008). It should be noted that our results do not exclude the possibility that C5L2 might be a multi-functional receptor, with different roles in different cell types. Moreover, it is also possible that human and rodent C5L2 have dissimilar functions although this is unlikely given the high sequence similarity between species. Mouse C5L2 lacks the ‘DRY’ and ‘NPXXY’ motifs and has a truncated third intracellular loop; the ligand specificity of mouse C5L2 is also analogous to human (Scola et al., 2007). Further work is clearly required to determine the range of activities of C5L2 and the role that this protein plays *in vivo*.

## Footnotes

2 T.M. Woodruff and C.R. MacKay, personal communication.
